# Research Misconduct in Low- and Middle-Income Countries

**DOI:** 10.1371/journal.pmed.1001315

**Published:** 2013-03-26

**Authors:** Joseph Ana, Tracey Koehlmoos, Richard Smith, Lijing L. Yan

**Affiliations:** 1 Calabar Women and Children Hospital, Calabar, Nigeria; 2 Faculty of Health Sciences, Cross River University of Technology, Calabar, Nigeria; 3 ICDDR,B, Dhaka, Bangladesh; 4 UnitedHealth Chronic Disease Initiative, London, United Kingdom; 5 The George Institute for Global Health at Peking University Health Science Center, Beijing, China

## Abstract

As part of a cluster of articles critically reflecting on the theme of “no health without research,” Richard Smith and colleagues lay out what is currently known about research misconduct in low- and middle-income countries, summarizing some high profile cases and making suggestions on ways forward.

**Summary Points**

- All human activity is associated with misconduct, and as scientific research is a global activity, research misconduct is a global problem.
- Studies conducted mostly in high-income countries suggest that 2%–14% of scientists may have fabricated or falsified data and that a third to three-quarters may be guilty of “questionable research practices.”
- The few data available from low- and middle-income countries (LMICs) suggest that research misconduct is as common there as in high-income countries, and there have been high profile cases of misconduct from LMICs.
- A comprehensive response to misconduct should include programmes of prevention, investigation, punishment, and correction, and arguably no country has a comprehensive response, although the US, the Scandinavian Countries, and Germany have formal programmes.
- China has created an Office of Scientific Research Integrity Construction and begun a comprehensive response to research misconduct, but most LMICs have yet to mount a response.

Research misconduct is a global problem as research is a global activity. Wherever there is human activity there is misconduct. But we lack reliable data on the extent and distribution of research misconduct, and few countries have mounted a comprehensive response to misconduct that includes programmes of prevention, investigation, punishment, and correction. The United States, the Scandinavian countries, and Germany have formal programmes [1], but even a country like the United Kingdom that has a long research tradition and has for years been debating research misconduct has failed to mount an adequate response [2]. But what of low- and middle-income countries (LMICs), many of which are investing heavily in research? There are some high profile cases of misconduct from these countries, but little has been published on research misconduct in LMICs. This article provides what might best be described as an initial sketch of research misconduct in LMICs. (Research misconduct has a specific definition, in the United States [see below], but we, like many others, use the term broadly in this paper to cover every kind of misconduct—major or minor and intentional or not.)

## Methods

We conducted a Medline search in June 2012 for studies and articles on research misconduct in LMICs and found little apart from reports of some cases. Several of us are part of a network of centres in LMICs conducting research on non-communicable disease, and we used this network to gather some information on research misconduct [3]. The centres are in China, Bangladesh, India, Tunisia, Kenya, South Africa, Mexico, Central America, Peru, and Argentina [3], and we sent a simple survey to the directors of these centres in these 11 centres asking about research misconduct. The simple questions we asked are shown in Box 1. We received answers from eight colleagues with our colleague from Guatemala answering for both his country and Costa Rica. In addition, one of us (JA) answered the survey for Nigeria. Table 1 summarises the answers, which are discussed later in the paper.

**Table 1:** Results to a survey of extent of and response to research misconduct in low- and middle-income countries.

Country	National System	Institutional System	Cases	Discussed
China	Yes, China Ministry of Science and Technology has a regulation effective Jan 1, 2007 (for science and technology programs)	Yes	Some	Some discussion, mostly by media on publicized cases
Southern cone (Argentina, Chile, Uruguay)	No	No	Some	No
Nigeria	No	No	High profile cases involving drug company, no local cases	Discussed by Nigerian editors
Tunisia	Yes, includes sanctions	No	No public cases	Beginning
Costa Rica	No, law proposed	No	No public cases	Yes, hence law proposed
Guatemala	Partial	No	High profile case of abuse by US researcher in 40 s	Yes, because of high profile cases
India	No	Yes	“Whispers” and case reported in BMJ and WSJ	Some, nothing formal
Peru	Partial	Yes, because NIH grant	No public cases. 3/30 masters students guilty of plagiarism	“Barely”
South Africa	Yes, National Ethics Council	Yes	One very high profile case. People aware that it happens	“Not much”
Bangladesh	No	Yes	Some cases reported	No

NIH, National Institutes of Health.

**Box 1. Questions Asked about Research Misconduct in Various Low- and Middle-Income Countries**

1. Does your country have a defined and effective national mechanism for responding to research misconduct?
2. If yes, can you describe it or provide a reference or URL?
3. Does your institution have a defined and effective mechanism for responding to research misconduct?
4. If yes, can you describe it or provide a reference or URL?
5. Can you tell us of cases of research misconduct in your country and/or institution (again you might provide references or a URL)?
6. Is the subject much discussed in your country?

In order to begin to sketch the problem of research misconduct in LMICs, we analysed the papers identified on research misconduct in LMICs and describe some of the cases mentioned by respondents to our survey, other high profile cases that are well known, and some cases that emerged from our search. This is not a systematic analysis of cases; rather the cases illustrate some of the issues. Nor are we prescribing a solution for LMICs, although clearly a comprehensive response should include prevention, investigation, punishment, and correction of the record.

## Defining Research Misconduct

Everybody agrees that research misconduct includes fabrication (making up results), falsification (manipulating processes and results), and plagiarism (stealing other's work). The US government has a long and specific definition of research misconduct [4], whereas Europeans have opted for shorter, general definitions [5]. For example, a recent joint meeting of BMJ and the Committee on Publication Ethics (COPE) reaffirmed an earlier definition of research misconduct as “Behaviour by a researcher, intentional or not, that falls short of good ethical and scientific standards.” [6].

What has become increasingly clear in recent years is that more damage may be done to the research record by “questionable research practices” than by the “big three” of fabrication, falsification, and plagiarism [7]. Questionable research practices include publishing pieces of research more than once, failing to declare conflicts of interest, excluding outlying data without disclosure (selective reporting), including on a paper an author who has contributed little or nothing, and many other things.

At the Second World Conference on Research Integrity in Singapore in 2010 the 341 attendees from 51 countries adopted the Singapore Statement, and it includes the following paragraph [8]:

“Researchers should report to the appropriate authorities any suspected research misconduct, including fabrication, falsification or plagiarism, and other irresponsible research practices that undermine the trustworthiness of research, such as carelessness, improperly listing authors, failing to report conflicting data, or the use of misleading analytical methods.”

“Irresponsible research practices” is the same as “questionable research practices.”

## How Common Is Research Misconduct?

Unsurprisingly it is difficult, probably impossible, to get reliable data on the prevalence and incidence of research misconduct, but Daniele Fanelli has conducted a systematic review and meta-analysis of surveys asking researchers about misconduct [9]. He was able to include 21 surveys in the systematic review and 18 in the meta-analysis and found that nearly 2% of scientists had themselves fabricated or falsified data and a third admitted to questionable research practices. (The review does not include data on plagiarism.) When asked about other researchers those surveyed said that 14% had fabricated or falsified data and nearly three-quarters were guilty of questionable research practices.

Fifteen of the studies were from the US, three from the UK, one from Australia, and two included people from more than one country; the most recent study gathered data in 2005. There was no study from an LMIC, and some studies are clearly needed.

Although we could find no systematic study of research misconduct in LMICs, there are studies that give some insights into the incidence of research misconduct in these countries.

A systematic review of studies of authorship provides some evidence that problems with authorship may be commoner in some LMICs than in the US or UK [10]. The authors identified 14 surveys reporting on misuse of authorship, and authorship misuse was reported by 55% (95% CIs 45%–64%) of responding researchers in four countries outside the US or UK compared with 23% in the USA or UK (95% CIs 18%–28%). Three of these studies came from LMICs with South Africa reporting a rate of 64% (95% CIs 44%–81%), India 38% (95% CIs 18%–62%), and Bangladesh 60% (95% CIs 44%–74%). We don't think that it would be legitimate to conclude confidently from this review of surveys of self reported problems with authorship that such problems are commoner in LMICs than in the US or UK, but the study shows that such problems do occur commonly in LMICs.

Another study of all the retractions of scientific studies from PubMed between 2000 and 2010 provides data on retractions from different countries [11]. Table 2 provides a ratio of studies retracted for fraud to total papers published for a series of selected studies, and the immediate conclusion is that the ratios are mostly similar—particularly, for example, between the US and all Asian countries, most of which are LMICs. There is some suggestion that India has a higher ratio, but it's not possible to conclude much from this study both because numbers are small and because it's probably only a small fraction of all fraudulent papers that are retracted. Again, however, this study confirms that LMICs do experience research misconduct.

**Table 2:** Ratio of retractions for fraud to total number of papers published for selected countries.

Country	Fraudulent Retractions 2000–2010	Number of Papers 2010 (Thousands)	Ratio of Fraudulent to Total Papers (Thousands)
USA	84	140	0.6
China	20	28	0.71
Japan	18	25	0.72
India	17	13	1.31
UK	7	41	0.17
Turkey	2	11	0.18
Iran	1	5	0.2
All Asian	63	94	0.67

An article discussing plagiarism in Latin American countries observes that nobody from Latin America attended the first World Conference on Research Integrity in 2006 and a search of 190,700 articles from Latin America in 594 journals in 2008 disclosed only ten articles on research misconduct [12]. The authors conducted focus groups with Brazilian researchers and found that people regarded copying text as much less serious than copying data. They found too that because of the pressure to publish in English language journals and the difficulty researchers had writing scientific English there was a great temptation to plagiarise text. The authors concluded that there should be formal guidance on research integrity and more training in writing scientific English.

There is discussion among editors of Western journals about plagiarism being common in articles submitted from China, which are growing rapidly, and an article in *Science* in 2006 wrote of China: “In this scientific Wild West, an unprecedented number of researchers stand accused of cheating—from fudging resumes to fabricating data—to gain fame or plum positions.” [13]. The articles do not, however, include data to back up this claim, but a letter in *Nature* in 2010 from the editor of the *Journal of Zhejian University-Science* reports that “a staggering” 31% of papers submitted to the journal included “unoriginal material.” [14]. The journal is prestigious within China and was the first to screen for plagiarism. The headline to the letter says “Chinese journal finds 31% of submissions plagiarised,” but much of the “unoriginal material” may have been referenced, meaning that it was not plagiarised.

In fact China has taken scientific integrity more seriously than many Western countries and certainly than any other LMIC [15]. Accusations of misconduct began to fly around China in the 1990s, but in 2005 the National Science Foundation of China investigated 542 allegations of misconduct and found evidence of misconduct in 60 cases. The main problems were data falsification (40%), plagiarism (34%), and data fabrication or theft (34%) [15]. After this investigation, the Ministry of Science and Technology took action and established the Office of Scientific Research Integrity Construction, which investigates allegations of misconduct. Various scientific bodies have published guidance on research integrity, and Peking University, the most prestigious in China, developed policies on research misconduct in 2001. Most other universities have followed. David Resnik and Weiqin Zeng argue in a comprehensive review of research integrity in China that China needs to go further with “promoting education in responsible conduct of research, protecting whistleblowers, and cultivating an ethical research environment.” [15].

China unlike many countries has recognised the problem at the highest level, and the Chinese premier Wen Jiabao has said “Our scientists need to cultivate scientific ethics; most importantly they need to uphold the truth, seek truth from facts, be bold in innovation and tolerant of failure. Only science and the spirit of seeking truth from facts can save China.”

## High Profile Cases in Low- and Middle-Income Countries

Action on research misconduct has been prompted in developed countries by high profile cases of misconduct, and the LMICs have had these—while some are historical and our review is not meant to be comprehensive, it's worth summarising some of them. The cases fall into two categories: misconduct by scientists native to the LMICs, and misconduct by researchers from high-income countries conducting research in the countries.

### South Africa

One of the recent tragedies of medicine was the widespread use of high dose chemotherapy and bone marrow transplantation in women with advanced breast cancer [16], and fraudulent trials from South Africa were crucial in promoting the treatment [16]. The hope was that the women could be given doses of chemotherapy high enough to kill the cancer cells and that they could avoid the toxicity of the drugs by being given autologous bone transplants. Bone marrow transplantation, as Siddhartha Mukherjee observes in his Pulitzer prize winning book, *The Emperor of all Maladies*, was “big business: big medicine, big money, big infrastructure, big risks.” [17].

Werner Bezwoda, an oncologist at the University of Witzwatersrand in Joahannesburg, was one of the leading transplanters with patients coming from three continents to be treated by him. His results were spectacular with 90% of patients achieving a complete remission from their cancer, as he described to a conference in San Diego in 1992. Nobody else managed to achieve such good results. Between 1991 and 1999 some 40,000 women with breast cancer were treated with bone transplantation at a cost of between US$2 billion and US$4 billion [17]. Randomised trials were hard to conduct because so many doctors and patients believed in the effectiveness of the treatment.

Nevertheless, at a major meeting in Atlanta in 1999 the results of four trials were presented. Bezwoda had once again achieved dramatic results with 60% of the patients in the treatment arm of his trial alive after 8 years compared with 20% in the control arm. But the other three trials had either equivocal or negative results [17].

The dramatic discrepancy in the results prompted serious doubts, and a team of American investigators went to South Africa to review Bezwoda's results. The investigators found multiple problems with Bezwoda's studies, including that many of the patients recorded as alive had actually been discharged for terminal care [16]–[18]. The ethics committee of the university had no record of the studies, and, very disturbingly, many of the patients were “barely literate black women” for whom consent forms could not be found, although Bezwoda reported having treated equal numbers of white women.

In February 2000 Bezwoda confessed to misconduct, resigned from his position, and effectively disappeared [17]. His studies were subsequently retracted, and very soon bone marrow transplantation ceased to be a treatment for women with advanced breast cancer.

As we tell this sad story, we wonder how Bezwoda could simply walk away from such misconduct. Perhaps this case is evidence for the idea that research should be regarded as a criminal offence, as has been recommended by Alexander McCall-Smith, a professor of law and medical ethics [19].

### India

Bezwoda may not have been convicted of misconduct, but his studies have been retracted and it's clear to the world that his work is fraudulent and should be disregarded. But another case from an LMIC, this time India, illustrates how difficult it can be to resolve cases of suspected misconduct [20].

RB Singh is a private practitioner based in Uttar Pradesh who has published dozens of papers in prestigious journals, including *BMJ*, *Lancet*, *Circulation*, and the *American Journal of Cardiology*. Between 1989 and 1993 he was the first author on 28 full articles, and he published five large intervention studies within 18 months [20]. In 1993 one of us (RS), then editor of the *BMJ*, was alerted to considerable anxieties about the work of Singh by several researchers.

When editors have doubts about scientific studies that they have either published or had submitted to them they must ask the employers of the researchers—often universities—to investigate. Employers, unlike editors, have the legal legitimacy to conduct an investigation and can ensure that due process is applied. Singh did not have an employer, so the *BMJ* tried asking several Indian organisations to investigate. But none of them could resolve the doubts, and in 2005 the *BMJ* took the unusual step of publishing an account of the whole saga [20].

Singh has always vigorously denied any misconduct and seen the *BMJ*'s doubts about his work having racist origins. Unable to prove or disprove research misconduct, both the *BMJ* and the *Lancet* published “expressions of concern” about the studies they had published by Singh [21],[22]. Although his work is ignored by many editors and researchers, Singh continues to publish.

This story illustrates the need to have national and even international mechanisms for investigating allegations of research misconduct. It's unsatisfactory for everybody that the body of medical evidence should contain so many studies about which there are severe doubts and that it remains unresolved whether RB Singh is guilty of research misconduct or has been falsely accused.

### China

In 2009 *JAMA* retracted a study from China in which the authors claimed in a randomised trial that transarterial chemoembolisation together with radiofrequency ablation therapy was better than either treatment alone in patients with large primary liver cancers [23]. Readers of *JAMA* raised concerns with the editors about the study, and after an investigation the editors contacted Shandong University, the institution of the main author. The university set up a panel to investigate and found that the study had not had ethical approval and was not a well done randomised trial and that “conclusions drawn from the study are not valid.” [22].

Three professors from Zheijang University lost their positions because of plagiarism [24]. He Haibo, an associate professor of pharmacology, is reported to have admitted to plagiarising or fabricating material in eight papers he submitted to international journals [24]. The newspaper, *China Daily*, wrote at the time: “This biggest-ever academic scandal is for sure a wakeup call that the Chinese universities are facing a crisis of credibility.” (Quoted in [24].)

### Guatemala

Guatemala, one of the countries included in our network, has experienced a case of research misconduct so severe that it led in 2011 to the president of the United States publicly apologising to the president of Guatemala [25]. Researchers from the United States, funded by the National Institutes of Health, between 1946 and 1948 without informed consent, infected around 1,500 soldiers, prostitutes, prisoners, and patients with mental health problems with syphilis and other sexually transmitted diseases. Some 83 people died directly from the experiments.

These experiments, which were not published, followed on from the infamous Tuskegee experiment in which between 1932 and 1972 poor black men in Alabama infected with syphilis were left untreated to see what would happen. The men were not told that they had syphilis [26].

The experiments in Guatemala did not come to light until they were discovered by Professor Susan Mokotoff Reverby of Wellesley College, who was researching the Tuskegee experiments. The experiments that were conducted in Tuskegee would never have been conducted among wealthier people in the US, and the follow-up experiments to Tuskegee were conducted in Guatemala because they could not, in the words of then Surgeon General in the US, be conducted domestically.

This case has led to great sensitivity about foreign researchers conducting research in Guatemala, all of Latin America, and, indeed, low- and middle-income countries.

### Nigeria

There is a longstanding anxiety that researchers from developed countries may conduct experiments in LMICs that would not be acceptable in developed countries. Marcia Angell, then an editor of the *New England Journal of Medicine*, published an influential article in her journal in 1997 arguing that researchers were still willing to conduct studies in developing countries that would not be acceptable in the US [27]. She used the example of using a placebo arm in a trial in Africa of trying to prevent vertical transmission of HIV when it was known that zidovudine had benefit in such circumstances. Others pointed out that the study had been approved in the US by several institutional review boards and argued that Angell failed to understand that there was no point in testing treatments that were unaffordable in Africa [28].

But some research is clearly unacceptable. In 2011 the drug company Pfizer paid US$175,000 compensation to families of children in Nigeria who had been involved in a trial conducted by the company in 1996 [29]. The study was conducted during a meningitis outbreak, and 200 children were given either trovafloxacin, a new drug from Pfizer, or ceftriaxone, standard treatment. The families of the children said that they were not told that their children were in a trial or that free effective treatment was available in the same hospital. Five of the children given trovafloxacin and six of those given ceftriaxone died. Pfizer also acknowledged in 2007 that some of the children had received only a third of the recommended does of ceftriaxone. Trovoflaxacin was later banned in Europe [29].

## Response to Research Misconduct in Low- and Middle-Income Countries

Table 1 shows that in most of the LMICs we examined, there appears to be little or no discussion of research misconduct and that most have no national body for dealing with misconduct. China is the one LMIC that has taken the problem most seriously by creating an Office of Scientific Research Integrity Construction to investigate allegations of misconduct and beginning a comprehensive response to research misconduct as we outlined in our introduction. But these actions have been in response to many accusations of misconduct [5], and they must go further [15]. It's not at all surprising that LMICs should not have national systems when so few developed countries don't either.

## What Now?

We join many others in arguing that all countries need national systems for responding to research misconduct. Most countries have systems for ethical approval of studies, but, in addition, countries need systems for preventing, investigating, punishing, and correcting research misconduct. The prime responsibility might lie with individual research institutions, usually universities, but even large research institutions in developed countries often lack the know-how to perform these functions adequately. Plus the institutions have profound conflicts of interest when one of their leading researchers is accused of research misconduct. A national body can supply support and leadership and help manage conflicts of interest.

There is an understandable tendency to deny research misconduct, and most countries seem to take decades of debate and exposure of cases of misconduct to accept the need for a systematic response. The first stage is discussion and recognition of the problem, and few LMICs seem to have reached even the stage of discussion. It may be, however, that the World Conference on Research Integrity, the third of which is to be held in Montreal in 2013, can shorten the process of establishing an effective response in LMICs, and bodies with experience, like the US Office of Research Integrity and the UK Research Integrity Office, can offer help. Each country, however, will need to work out its own system.

## Abbreviations

LMIC: low- and middle-income country

## References

[1] Tavare A (2011) Managing research misconduct: is anyone getting it right? BMJ 343: 2821d. 10.1136/bmj.d8212 22207043

[2] Godlee F, Wager E (2012) Research misconduct in the UK. BMJ 344: d8357. 22218103 10.1136/bmj.d8357

[3] NIH (2009) UnitedHealth and NHLBI Centers of Excellence. Available: http://www.nhlbi.nih.gov/about/globalhealth/centers/index.htm. Accessed 20 August 2012.

[4] Public Health Service policies on research misconduct. Final rule. Fed Regist 70: 28369–28400. 15898182

[5] Smith R (2000) What is research misconduct? J Roy Coll Physicians Edin 30: 4–8.

[6] A Consensus Statement on Research Misconduct in the UK, BMJ/COPE High Level Meeting 12 January 2012. Available: http://publicationethics.org/files/A_consensus_statement_on_research_misconduct_in_the_UK. Accessed 17 December 2012.

[7] Martinson BC, Anderson MS, de Vries R (2005) Scientists behaving badly. Nature 435: 737–738. 15944677 10.1038/435737a

[8] (2010) Singapore Statement on Research Integrity. 2nd World Conference on Research Integrity; 21–24 July 2010; Singapore. Available: http://www.singaporestatement.org/statement.html. Accessed 20 August 2012.

[9] Fanelli D (2009) How Many scientists fabricate and falsify research? a systematic review and meta-analysis of survey data. PLoS ONE 4 (5) e5738 doi:10.1371/journal.pone.0005738. 19478950 10.1371/journal.pone.0005738 PMC2685008

[10] Marušić A, Bošnjak L, Jerončić A (2011) A systematic review of research on the meaning, ethics and practices of authorship across scholarly disciplines. PLoS ONE 6 (9) e23477 doi:10.1371/journal.pone.0023477. 21931600 10.1371/journal.pone.0023477 PMC3169533

[11] Steen RG (2011) Retractions in the scientific literature: do authors deliberately commit research fraud? J Med Ethics 37: 113–117. 21081306 10.1136/jme.2010.038125

[12] Vasconcelos S, Let J, Costa L, Pinto A, Sorenson MM (2009) Discussing plagiarism in Latin American science. EMBO Reports 10: 677–682. 19525923 10.1038/embor.2009.134 PMC2727439

[13] Xin H (2006) Scandals shake Chinese science. Science 312: 1464–1466. 16763127 10.1126/science.312.5779.1464

[14] Zhang Y (2010) Chinese journal finds 31% of submissions plagiarized. Nature 467: 153. 10.1038/467153d 20829776

[15] Resnik D, Zeng W (2010) Research integrity in China: problems and prospects. Dev World Bioeth 10: 164–171. 19832885 10.1111/j.1471-8847.2009.00263.x PMC2891906

[16] Welch HG, Mogielnicki J (2002) Presumed benefit: lessons from the American experience with marrow transplantation for breast cancer. BMJ 324: 1088–1092. 11991918 10.1136/bmj.324.7345.1088 PMC1123033

[17] Mukherjee S (2011) The emperor of all maladies. London: Fourth Estate. 592 p.

[18] Weiss RB, Rifkin RM, Stewart FM, Theriault RL, Williams LA, et al (2000) High-dose chemotherapy for high-risk primary breast cancer: an on-site review of the Bezwoda study. Lancet 355: 999–1003. 10768448 10.1016/S0140-6736(00)90024-2

[19] McCall-Smith A (2000) What is the legal position? J Roy Coll Surg Edin 30: 22.

[20] White C (2005) Suspected research fraud: difficulties of getting at the truth. BMJ 331: 281–288. 16052022 10.1136/bmj.331.7511.281 PMC1181273

[21] Expression of concern. BMJ 331: 266. 10.1136/bmj.331.7511.266 PMC1181266 16052018

[22] Horton R (2005) Expression of concern: Indo-Mediterranean Diet Heart Study. Lancet 366: 354–356. 16054927 10.1016/S0140-6736(05)67006-7

[23] DeAngelis CD, Fontanarosa PB (2008) Retraction: Cheng B-Q, et al. Chemoembolization combined with radiofrequency ablation for patients with hepatocellular carcinoma larger than 3 cm: a randomized controlled trial. JAMA 299: 1669–1677. JAMA 2009 301: 1931. 10.1001/jama.2009.640 19380477

[24] Ford P (2009 March 23) China targets an academic culture of cut-and-paste. Christian Science Monitor

[25] Tanne JH (2010) President Obama apologises to Guatemala over 1940s syphilis study. BMJ 341: c5494. 20921075 10.1136/bmj.c5494

[26] Jones JH (1981) Bad blood: the Tuskegee syphilis experiment. New York City: Free Press.

[27] Angell M (1997) The ethics of clinical research in the third world. N Engl J Med 337: 847–849. 9295243 10.1056/NEJM199709183371209

[28] Halsey NA, Sommer A, Henderson DA, Black RE (1997) Ethics and international research. BMJ 315: 965–966. 9365287 10.1136/bmj.315.7114.965 PMC2127643

[29] Lenzer J (2011) Pfizer settles with victims of Nigerian drug trial. BMJ 343: d5268. 21846712 10.1136/bmj.d5268

